# The relevance of RNA–DNA interactions as regulators of physiological functions

**DOI:** 10.1007/s00424-025-03091-7

**Published:** 2025-05-21

**Authors:** Julia Stötzel, Timothy Warwick, Praveenya Tirunagari, Ralf P. Brandes, Matthias S. Leisegang

**Affiliations:** 1 Institute for Cardiovascular Physiology, Goethe University, Frankfurt, Germany; 2 German Centre of Cardiovascular Research (DZHK), Partner Site RheinMain, Frankfurt, Germany

**Keywords:** R-loop, RNA–DNA triplex, Physiology, Gene regulation, Chromatin dynamics

## Abstract

RNA–DNA interactions are fundamental to cellular physiology, playing critical roles in genome integrity, gene expression, and stress responses. This review highlights the diverse structures of RNA–DNA hybrids, including R-loops, RNA–DNA triplexes, and RNA–DNA hybrid G-quadruplexes (hG4s) and their relevance in physiology. R-loops are formed during transcription and replication, which regulate gene expression and chromatin dynamics but can also threaten genome stability. RNA–DNA triplexes, often formed by long noncoding RNAs (lncRNAs) such as *FENDRR* and *MEG3*, recruit chromatin modifiers like Polycomb repressive complex 2 to modulate gene expression, influencing organogenesis and cell specification. hG4s, formed by guanine-rich sequences in RNA and DNA, regulate transcription termination and telomere stability. Through this, hG4s can affect gene suppression and replication regulation. RNA–DNA hybrids are tightly regulated by helicases, RNase H enzymes, and topoisomerases, with altered regulation linked to genomic instability and disease. This review discusses the complexity of RNA–DNA interactions and their recently identified contributions to cellular physiology.

## Different types of RNA–DNA interactions are existing

**Fig. 1:**
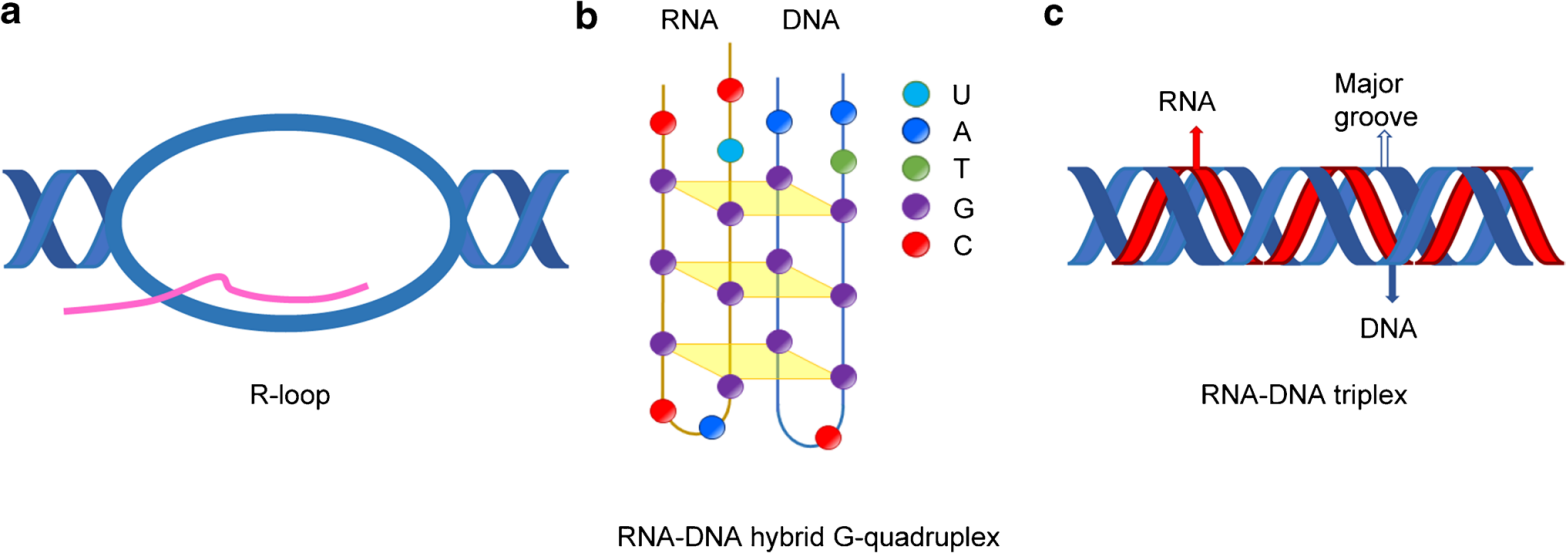
RNA can bind DNA in many different ways, leading to structures such as R-loops, RNA–DNA hybrid G-quadruplexes, or RNA–DNA triplexes. **a** R-loops are three-stranded structures that occur when an RNA molecule (pink) hybridizes with the DNA template strand (blue), displacing the non-template DNA strand. **b** RNA–DNA hybrid G-quadruplex structures are non-canonical nucleic acid structures comprising stacked guanine tetrads. **c** Triplexes are formed when a single-stranded RNA strand (red) integrates into the major groove of double-stranded DNA (blue) through Hoogsteen base pairing

Nowadays, RNA–DNA interactions are emerging as fundamental regulatory elements in cellular processes that govern genome integrity, gene expression, and cellular response to stress [56]—a paradigm shift rooted in a century of foundational discoveries about nucleic acids. While DNA (originally termed “nuclein”) was discovered in the nineteenth century by Friedrich Mieschner [18], the twentieth century brought groundbreaking discoveries, including the double helix structure of DNA by Watson and Crick [75], building on Rosalind Franklin’s X-ray crystallography [24]. Felsenfeld’s observation of three-stranded poly(A)-poly(U) structures [21] and Gellert’s description of G-tetrads further revealed the complexity of nucleic acid interactions [29]. Karst Hoogsteen’s discovery of an alternative hydrogen-bonding pattern in 1963 [34], now known as Hoogsteen base pairing, provided a structural basis for RNA–DNA triplexes and other non-canonical structures. Ronald Davis and colleagues (1976) identified R-loops [66], and since then, detailed characterizations have followed. Separately, computational tools have deepened our understanding of RNA–DNA interactions [74]. While much of the research in molecular biology focused on the canonical role of RNA in DNA transcription and mRNA translation, an increasing body of evidence suggests that RNA–DNA interactions are more diverse and physiologically significant than previously appreciated. These interactions encompass a variety of structural forms, such as R-loops (Fig. 1a), RNA–DNA hybrid G-quadruplexes (Fig. 1b), and RNA–DNA triplexes (Fig. 1c). These elements are critical for the regulation of transcription, chromatin remodeling, DNA repair, and telomere maintenance [44, 56, 93]. Despite growing evidence for the physiological importance of these RNA–DNA interactions, significant knowledge gaps persist.

R-loops: One of the most studied form of RNA–DNA interactions are R-loops. These are three-stranded nucleic acid structures consisting of an RNA–DNA hybrid and a displaced single-stranded DNA [56]. R-loops occur genome-wide and are found in many organisms, from bacteria to humans [85]. They contribute to gene expression in several ways. In some cases, R-loops lead to transcriptional silencing and the induction of repressive chromatin marks, resulting in impaired gene expression [62]. They can, however, also act as transcriptional activators involving head-to-head antisense transcription, as shown at the Vimentin locus [8]. While required for normal cellular functions, R-loops can also lead to genomic instability when improperly regulated. The consequence of this genomic instability is DNA damage and the initiation of diseases such as cancer and neurodegenerative disorders [56]. The dynamic formation and resolution of R-loops are tightly controlled, with a range of proteins involved. Among them are RNA-binding proteins, helicases, topoisomerases, nucleases, and chromatin modifiers. These orchestrate the delicate balance between RNA and DNA and also interfere with genomic stability [10, 27, 56, 85]. For example, RNase H resolves R-loops by cleaving the phosphodiester bonds of RNA in RNA–DNA hybrids, while leaving RNA within RNA–DNA triplexes intact [12, 44, 53].

RNA–DNA triplexes and RNA–DNA hybrid G-quadruplexes: In addition to the well-characterized R-loops, more complex structures such as RNA–DNA triplexes and RNA–DNA hybrid G-quadruplexes are becoming increasingly recognized for their roles in regulating gene expression and maintaining genomic integrity. RNA–DNA triplexes, which are formed by sequence-specific binding of RNA to the major groove of double-stranded DNA through Hoogsteen-base pairing, are primarily involved in gene regulation [44]. These structures can bind transcription factors and chromatin-modifying complexes to target genes that affect their transcriptional outcomes. RNA–DNA hybrid G-quadruplexes (hG4s), composed of guanine tracts from both a nontemplate DNA strand and an RNA transcript, have been observed near transcriptional start sites (TSS) [78, 88]. Interestingly, hG4s were also found at telomeres, where they may play a role in maintaining chromosomal integrity by protecting the 3′end of the telomeric DNA overhang, which is critical for the interaction with telomerase and other telomere-associated proteins [82].

Understanding the roles of RNA–DNA interactions for cellular physiology offers promising avenues for therapeutic intervention, particularly in diseases associated with genomic instability and alterations of gene expression. For example, pathological R-loop formation has been implicated in cancer. Here, R-loops contribute to oncogene activation or tumor suppressor gene silencing. Neurodegenerative diseases and defective RNA processing trigger R-loop accumulation, which results in disease progression [56]. Moreover, RNA–DNA interactions are increasingly being explored for their potential in gene regulation strategies, such as for CRISPR/Cas-based gene editing applications [55].

This review focuses on the different kinds of RNA–DNA interactions and their diverse roles during cellular physiology, from transcriptional regulation to chromatin remodeling and telomere maintenance. We will explore the diverse types of interactions, discuss the role of specific RNA molecules, and highlight the challenges and future directions in this exciting field of research.

## R-loops are dynamic structures relevant for physiology and pathophysiology

**Fig. 2:**
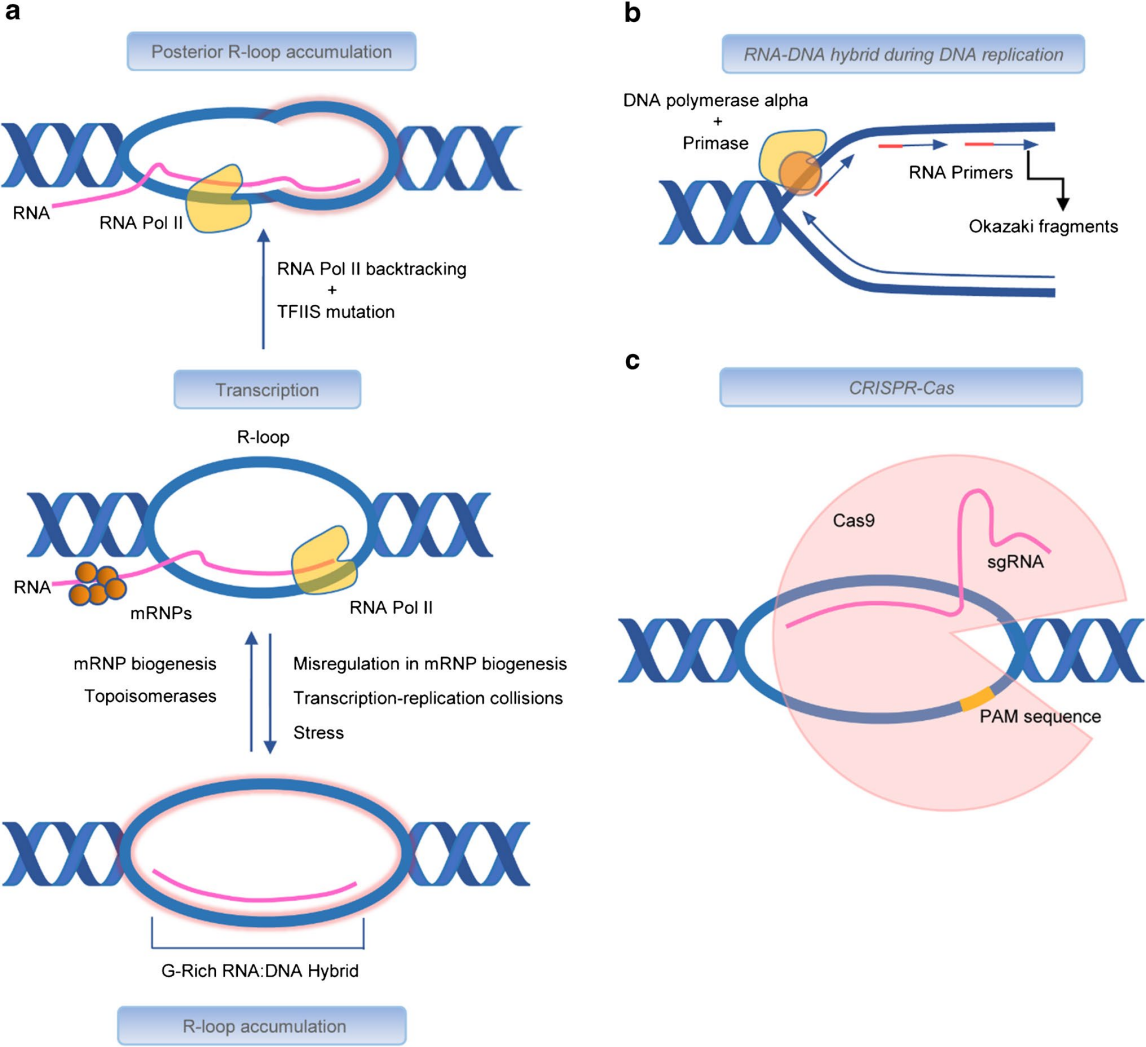
Mechanisms of R-loop and RNA–DNA hybrid formation during different biological processes. **a** R-loops are formed co-transcriptionally with many different proteins contributing. Stress, messenger ribonucleoprotein (mRNP) misregulation, and transcription-replication collisions can lead to R-loop accumulation, whereas RNA Pol II backtracking and mutation in the elongation factor TFIIS can lead to posterior R-loop accumulation. **b** RNA–DNA hybrids during DNA replication. **c** R-loop formation by single guide-RNA (sgRNA) pairing to target DNA in CRISPR-Cas genome editing processes

RNA–DNA hybrids are essential components of fundamental cellular processes such as transcription, replication, and genome regulation. These hybrids, including structures like R-loops, play vital roles in maintaining cellular function, genomic stability and are part of modern biotechnological techniques (Fig. 2a–c).

### Transient RNA–DNA hybrids during transcription and replication

During transcription, RNA polymerase II (Pol II) transiently forms RNA–DNA hybrids within the transcription bubble. The mechanism of Pol II is based on a forward translocation (Brownian ratchet) at the DNA template, leading to transcription elongation [41]. Here, the Pol II core enzyme, nascent RNA, and dsDNA form the ternary elongation complex (TEC). Pol II synthesizes nascent RNA by incorporating nucleoside triphosphates (NTPs) complementary to the DNA template [1, 41, 52]. The resulting short RNA–DNA duplex (~ 10 nucleotides) within the TEC stabilizes transcription elongation [1, 41, 52]. Using cryo-electron microscopy, it was shown that the nascent RNA detaches from the hybrid upstream and is released through a specific RNA exit tunnel in yeast (possibly via the Pol II core complex subunits Rpb4 and Rpb7) [13, 68]. The transcription bubble encompasses one or two nucleotides before the 3′end of the RNA until the two DNA strands anneal almost immediately after the hybrid [5]. Thus, the formation of these R-loops displaces the second DNA strand, resulting in the typical three-stranded structure [27, 52, 56].

In addition to the previously described R-loops, transient re-annealing of the nascent RNA to the DNA template as it exits RNA polymerase (thread back model) has been shown to form additional co-transcriptional R-loops immediately after the transcription bubble [56]. This can be observed when the packaging of nascent RNA into messenger ribonucleoprotein particle (mRNPs) is disrupted [61].

Pol II dynamics, including backtracking, where Pol II moves backwards, dissociating the catalytic site from the 3′end of the RNA and leading to elongation stress and arrest [54], exacerbate R-loop accumulation increasing the risk of transcription-replication collisions (TRCs) and DNA damage [35, 54, 56, 61, 87]. For example, Zatreanu et al. investigated mutations in transcription elongation factor S-II (TFIIS) in human cells (HEK293 T-REx and U2OS cells) and identified R-loop accumulation posterior of Pol II as well as newly discovered R-loop formation anterior of Pol II indicating that backtracking may lead to direct RNA–DNA hybrid accumulation [87] (Fig. 2a).

Cellular mechanisms regulate R-loop resolution through the action of key enzymes. Helicases like senataxin (SETX), BLM RecQ Like Helicase (BLM), RecQ Like Helicase 5, and RNase H enzymes or topoisomerases resolve RNA–DNA hybrids to prevent genomic instability [25–27, 85]. Dysfunction of these processes is linked to transcriptional interference, stalled replication forks, and human diseases such as neurodegeneration and the Aicardi-Goutières syndrome (AGS) [16, 58]. AGS is a progressive genetic encephalopathy triggered by overproduction of type I interferon caused by specific mutations in genes important for nucleic acid metabolism, like the RNA degrader RNase H [33].

RNA–DNA hybrids are critical during lagging strand replication. Short RNA primers, synthesized by the Pol α-primase complex, initiate replication at Okazaki fragments [46] (Fig. 2b). Pol α extends these primers with short DNA sequences before DNA polymerase δ takes over, synthesizing DNA discontinuously [46]. Flap structure-specific endonuclease 1 and RNase H2 remove RNA primers, and DNA ligase 1 ligates the fragments to form a continuous strand [11, 56].

Despite the high precision of the replication process, nucleotides can be misincorporated. Thus, RNA–DNA hybrids arise as a consequence of a faulty mechanism of replicative DNA polymerase, which misincorporates a ribonucleotide instead of a deoxyribonucleotide due to the nuclear abundance of ribonucleotide triphosphates compared to deoxynucleotide triphosphates [14, 47, 51]. Misincorporation threatens DNA stability and structural integrity, necessitating removal by RNase H2 via ribonucleotide excision repair [12, 40, 64]. The critical role of RNase H2 is underscored by its lethality when absent in mice and its association with genomic instability and disease [17, 58].

### Functions of R-loops in cellular physiology

**Fig. 3:**
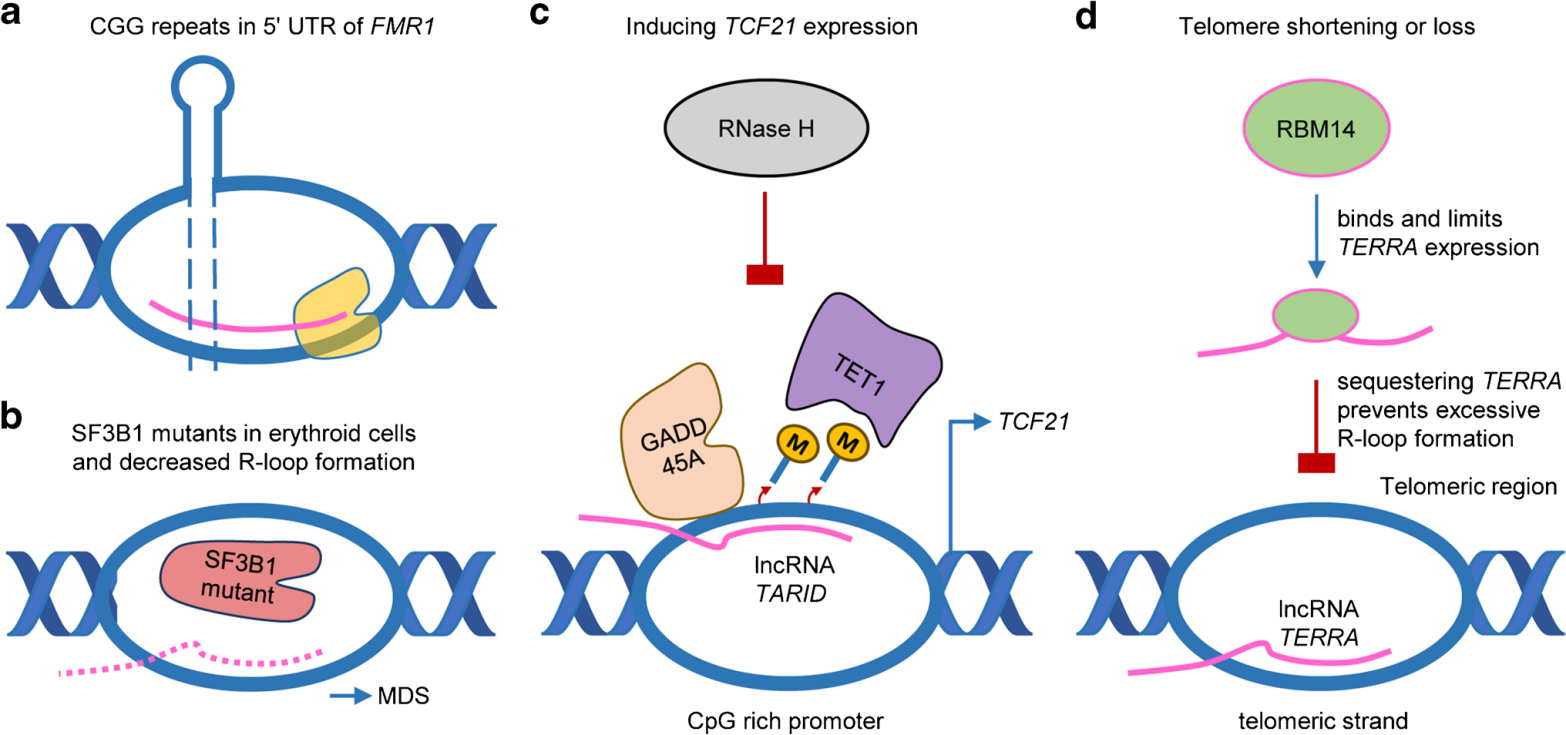
Examples of individual R-loops. **a** Co-transcriptional R-loop formation occurs at the 5′UTR of the CGG repeat region of the *FMR1* gene contributing to a number of heritable disorders. **b** In erythroid cells, mutations of the splicing factor SF3B1, which are associated with myelodysplastic syndromes (MDS), lead to decreased R-loop formation, intron retention reduction and deregulated gene expression. **c** R-loop formation at the tumor suppressor gene *TCF21* mediated by its antisense lncRNA *TARID* is recognized by GADD45A, recruiting TET1 to induce DNA demethylation of the *TCF21* promoter. This leads to an upregulation of TCF21, while RNase H, which degrades RNA within RNA–DNA hybrids, inhibits this process. **d** The lncRNA *TERRA*, which is regulated by RBM14, forms an R-loop at telomeric regions facilitating the elongation of short telomers. However, excessive accumulation of R-loops leads to replication stress and affects genome stability

R-loops form dynamically during transcription and replication, depending on RNA sequence and structure. For example, RNAs with long 5′-untranslated regions (UTRs) are prone to R-loop formation, because these regions are often GC-rich and do not form stable secondary structures. An example includes the human fragile X mental retardation 1 gene (*FMR1*), which has a CGG repeat element in the 5′UTR of the gene and has been implicated in the pathogenesis of several genetic disorders (Fig. 3a) [19, 45]. Use of the RNA–DNA hybrid antibody S9.6 facilitated immunoprecipitation, and chemical single-stranded DNA footprinting assays revealed co-transcriptional R-loop formation at the *FMR1* locus. This R-loop increased in length and complexity with upregulated transcription and expanded CGG repeats [45]. Another evidence for the importance of 5′UTR associated R-loop formation came from studies about splicing factor 3b subunit 1 (*SF3B1*). Here, mutants of *SF3B1* have been shown to induce a significant loss of R-loops in erythroid cells at the 5’UTR, whereas R-loops at 3′UTRs and intergenic regions remained. This effect was seen in parallel to splicing defects, such as a reduction of intron retention. As patients with the myelodysplastic syndrome (MDS) with mutated *SF3B1* suffer from bone marrow ring sideroblasts and ineffective erythropoiesis, Rombaut et al. demonstrated that mutated *SF3B1* leads to increased immature erythroblasts proliferation and lower ability for terminal differentiation, linking MDS to R-loop formation [60] (Fig. 3b).

LncRNAs also generate R-loops with regulatory roles. For example, the antisense lncRNA *TARID* is able to generate an R-loop at the *TCF21* promoter. Binding of growth arrest and DNA damage protein 45 A (GADD45A) to the R-loop recruits the DNA demethylation factor Ten-Eleven translocation 1 (TET1), which results in DNA demethylation of the *TCF21* promoter and the activation of *TCF21* transcription [2] (Fig. 3c). R-loops can also be formed by telomeres. Telomeres are essential for the stability and functionality of cells by protecting the ends of chromosomes. In particular, the enzyme telomerase and its protein subunit telomerase reverse transcriptase (TERT) are involved in the protection of the chromosome from DNA loss through the synthesis of new telomeric repeats [7, 63]. The lncRNA telomeric repeat-containing RNA (*TERRA*) is known to be associated with telomere maintenance and R-loop formation at telomeres [3, 23, 80]. However, excessive R-loop formation can lead to replication stress, chromosomal instability, and telomere loss. This can be observed in the context of RNA Binding Motif Protein 14 (RBM14) deficiency, as RBM14 appears to regulate *TERRA* levels and stability [72] (Fig. 3d). Proteins like Splicing Factor Proline And Glutamine Rich (SFPQ) and Non-POU domain-containing octamer-binding protein (NONO) modulate *TERRA*-associated R-loops, influencing telomeric replication and repair [57].

R-loops are implicated in ribosomal RNA (rRNA) transcription and nucleolar homeostasis. Decreased SETX levels lead to a promoter and pre-rRNA antisense (*PAPAS*)-dependent increase in repair factor replication protein, resulting in nucleolar relocalization. These conditions also affect rRNA genes, which are subjected to massive R-loop formation [22]. Of note, SETX is a RNA/DNA helicase resolving R-loops by catalyzing the unwinding of the RNA-DNA hybrid portion of the R-loops. Mutations of SETX have been associated with neurological disorders and cancer [28]. While lncRNAs have received considerable attention, other non-coding RNAs such as circular RNAs (circRNAs) have also been shown to form R-loops under certain conditions, potentially affecting their regulatory functions. Although shown in *Arabidopsis* plants, circRNAs can form R-loops with their parent genes, potentially influencing their function in various cellular processes [15]. It was shown that *circSMARCA5*, a circRNA derived from the chromatin remodeling protein SMARCA5, can potentially form an R-loop with its parent gene, as demonstrated by anti-S9.6 dot blot and RNase H treatment. The authors concluded that *circSMARCA5* inhibits the expression of SMARCA5 and decreases the DNA repair capacity in MCF-7 cells [81].

Alternative RNA splicing is a mechanism driven by splicing factors to generate different variants of RNA and proteins from the same gene [49]. An example of one of these splicing factors is the tumor suppressor MEN1, which is thought to be involved in the regulation of alternative precursor mRNA splicing. MEN1 induces a slowing of Pol II elongation, preventing exon skipping and thus the accumulation of R-loops, thereby limiting the associated DNA damage and genomic instability [38].

In conclusion, R-loops represent the most well-characterized class of RNA–DNA interactions with a plethora of physiological functions but also a substantial disease relevance.

## RNA–DNA triplexes fine-tune physiological gene expression

**Fig. 4:**
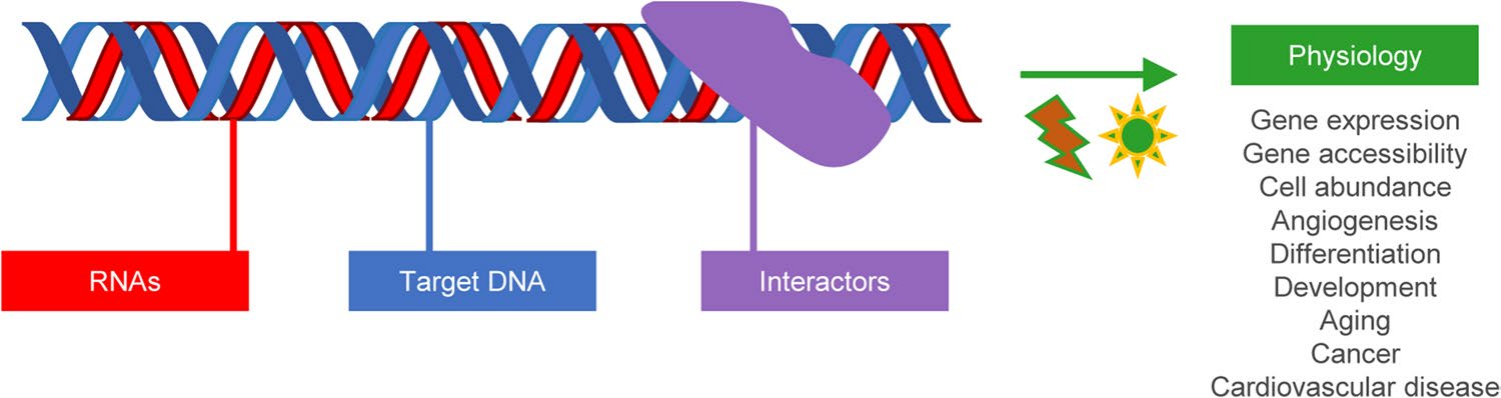
RNA–DNA triplexes and their physiological consequences. Multiple RNAs (primarily lncRNAs) have been studied for their ability to form triplexes with their respective target DNA sequences. The target DNA can be within promoters, exons, introns, 5′UTR, enhancers, or repetitive elements. The RNA-DNA triplexes formed potentially facilitate binding and interaction with key molecular interactors such as chromatin remodelers and transcription factors leading to important physiological and pathophysiological consequences

RNA–DNA triplexes are formed when a single-stranded RNA binds to the major groove of a double-stranded DNA through Hoogsteen or reverse Hoogsteen hydrogen bonds, typically at purine-rich regions of the DNA. These structures often involve lncRNAs and play critical roles in gene regulation, chromatin organization, and cellular homeostasis. Although their biological functions were initially enigmatic, recent advances in biophysical, biochemical, and computational tools have shed light on their physiological significance. Triplexes appear to influence gene expression by recruiting chromatin modifiers or transcription factors to specific loci, either repressing or enhancing transcription [44, 74] (Fig. 4). Biophysical techniques along with biochemical methods including RNase H and computational tools like Triplexator, Triplex Domain Finder, and TriplexAligner have been instrumental in identifying and characterizing RNA–DNA triplexes [44, 74]. Below, we discuss key examples of RNA–DNA triplexes and their physiological roles, supported by experimental evidence.

The lncRNA *FENDRR* (FOXF1 Adjacent Non-Coding Developmental Regulatory RNA) exemplifies the role of RNA–DNA triplexes in organogenesis. *FENDRR* forms RNA–DNA triplexes with the promoter regions of genes such as Foxf1 and Pitx2, which are involved in lateral mesoderm differentiation. Triplex formation leads to the recruitment of the Polycomb Repressive Complex 2 (PRC2) to these loci, resulting in epigenetic repression that silences transcription. Thereby, *FENDRR*-mediated gene regulation is critical for proper heart and lung development [31, 32].

The lncRNA hypoxia-inducible factor-1-alpha-antisense 1 (*HIF1α-AS1*), which is downregulated in certain pathological conditions, forms RNA–DNA triplexes at the promoter region of the pro-angiogenic gene *EPHA2*. Through this triplex, *HIF1α-AS1* recruits the human silencing hub (HUSH) complex member MPP8 and the histone methyltransferase SETDB1, leading to H3K9me3-mediated heterochromatin formation and transcriptional repression. This mechanism not only silences *EPHA2* but also contributes to *HIF1α-AS1*’s pro-apoptotic and anti-proliferative effects in various cellular contexts. Experimental studies have identified exon 1 of *HIF1α-AS1* as a critical region for mediating chromatin interactions and gene silencing [43]. These findings link *HIF1α-AS1* to cellular stress responses and its potential involvement in diseases such as pulmonary hypertension, cancer, and vascular disorders [30, 43, 71, 76, 92].

*SARRAH* (Senescence-Associated Regulatory RNA in Heart) is a lncRNA playing a pivotal role in maintaining cardiac health, particularly during aging. *SARRAH* forms RNA–DNA triplexes with specific cardiac gene loci. It recruits transcriptional coactivator p300 and the cysteine-rich protein 2, enabling transcriptional activation. *SARRAH* promotes cardiomyocyte survival, enhances contractility, and reduces apoptosis, as also demonstrated in mouse models of myocardial infarction. *SARRAH*’s anti-apoptotic properties diminish with age, potentially contributing to age-related cardiac dysfunction. The therapeutic potential of *SARRAH* to combat cardiac aging and improve post-injury recovery highlights its significance in cardiovascular biology [67]. 

The lncRNA *KCNQ1OT1* plays a critical role in maintaining genome stability and preventing cellular senescence by forming RNA–DNA triplexes with repetitive DNA elements, guiding epigenetic silencing through DNA methylation and H3K9me3 marks. Disruption of *KCNQ1OT1* or its repeat-rich region leads to transposon activation, heterochromatin reorganization, and cellular senescence [91].

One of the first lncRNAs studied in relation to RNA–DNA triplex formation was the lncRNA *MEG3* (Maternally Expressed Gene 3). *MEG3* forms RNA–DNA triplexes at the promoter regions of genes involved in the TGF-β pathway. *MEG3* recruits PRC2 and other chromatin modifiers to these loci, inducing H3K27me3 and transcriptional repression. The study by Mondal et al*.* was performed in BT-549, a human breast cancer cell line, and downregulation of *MEG3* led to increased invasion into matrigel, which was reversed in the presence of an TGF-β2 inhibitor [50]. In addition, Krause et al. used biophysical methods to distinguish between RNA–DNA heteroduplexes and triplexes, providing additional biophysical evidences that the above mentioned *FENDRR* and *MEG3* lncRNAs can form triplexes [42].

GALNT8 Antisense Upstream 1 (*GAU1*) is a lncRNA driving gastric cancer tumorigenesis by forming an RNA–DNA triplex with the promoter of the oncogene Sentrin-specific protease 5 (*SENP5*). The peptidylprolyl isomerase PPIA is central due to its recruitment of *GAU1* to the *SENP5* promoter, leading to epigenetic rearrangements that favor *SENP5* transcription, revealing a novel mechanism of isomerase-assisted lncRNA–DNA hybridization in cancer progression [90]. Regenerating Family Member 1 Gamma (*REG1CP*) is another lncRNA involved in cancer. Yari et al. showed that *REG1CP* promotes cancer cell cycle progression and tumorigenicity through facilitating transcription of Regenerating Family Member 3 Alpha (*REG3A*). Mechanistically, *REG1CP* forms an RNA–DNA triplex distal to the *REG3A* TSS and recruits the helicase Fanconi anemia complementation group J (FANCJ) to the promoter, where it unwinds DNA and relieves transcriptional repression. This mechanism enables glucocorticoid receptor α (GRα)-mediated activation of *REG3A* transcription, establishing *REG1CP* as a critical regulator of enhancer complex assembly and gene expression [86]. Another lncRNA, cisplatin-sensitivity-associated lncRNA (*CISAL*), is involved in cisplatin sensitivity in tongue squamous cell carcinoma (TSCC). *CISAL* forms an RNA–DNA triplex at the BRCA1 DNA Repair Associated (*BRCA1*) promoter, which leads to *BRCA1* transcriptional inhibition and promotion of mitochondrial fission. Interestingly, Fan et al. showed that high *CISAL* and low *BRCA1* levels are associated with longer survival times in TSCC patients [20].

The examples presented here illustrate the diverse physiological and molecular roles of RNA–DNA triplexes, ranging from organogenesis to cancer and stress responses. While these structures were ignored for a long time, RNA–DNA triplexes are now emerging as an additional layer of gene regulation. A more recently characterized type of RNA–DNA interaction occurs through RNA–DNA hybrid G-quadruplexes, which have also been linked to several areas of cellular physiology.

## RNA–DNA hybrid G-quadruplexes and G-quadruplex-telomere RNA structures

Other nucleic acid secondary structures are three-dimensional G-quadruplexes (G4), which are formed by guanine-rich sequences. G4s exist in DNA and RNA and are composed of four guanine bases arranged in a square-planar conformation and connected by Hoogsteen hydrogen bonds. This structure is further stabilized by a central cation such as K^+^. G4s are located in gene regulatory regions and are critical for cellular processes such as transcription, translation, telomere function, and genomic modifications [59, 69]. Latest progress in the study of G4s revealed that RNA–DNA hybrid G4s (hG4s) also form in living cells [4, 69, 78, 88].

hG4s appear to be involved in gene regulation processes such as transcription with a potential role in transcription regulation. During transcription, a co-transcriptional R-loop appears to be formed first [77]. Wanrooj et al. found a hG4 structure formed between the RNA transcript and the non-template DNA strand at the conserved sequence block II (*CSBII*) locus in human mitochondria. This hG4 promotes transcriptional termination as it potentially hinders the mitochondrial replication machinery to initiate DNA synthesis because of the removal of the RNA primer’s 3′end from the template strand making the primer inaccessible for the replication machinery. Thereby, hG4 potentially determine the DNA synthesis in human mitochondria [73]. Additionally, Zhang et al. investigated the *NRAS* locus and found also that formation of hG4 involved G-tracts from both the non-template DNA strand and RNA transcript resulting in gene suppression [93]. Zhang et al. found in a follow-up study that after transcription and co-transcriptional R-loop formation, the nascent RNA is released after the next round of transcription as a single-stranded RNA, allowing two G-tracts of this RNA to form an RNA–DNA G4 with two of the G-tracts of the template DNA [89]. Additionally, hG4s with two G-quartets were also identified near multiple TSSs, which show reduced stability and flexibility. At the same time, their folding processes show a higher sensitivity to transcriptional activity, associated with a higher occurrence of the potential RNA–DNA hybrid quadruplex-forming sequence on the non-template strand at the downstream side of TSSs [78]. A hG4 was also detected upstream of the TSS. Its formation was dependent on negative supercoiling, which is induced by the RNA polymerase [88].

As mentioned above, telomeres protect the ends of chromosomes [7, 63]. The protein–DNA complexes at the chromosome ends contain the double-stranded repetitive sequence 5′-TTAGGG-3′ and are also known to form G4s. The G4 binding structures at telomeres include telomeric proteins (RIFI), telomeric repeat-binding factor 2 (TRF2), and the lncRNA *TERRA* [69]. In addition to the aforementioned hG4 structures involved in transcription, hG4s have also been detected at telomeres. First, Xu et al. carried out several in vitro studies. In a first attempt, a hG4 derived from human telomeric DNA and RNA sequences was detected by nuclear magnetic resonance (NMR) spectroscopy using copper-catalyzed azide-alkyne cycloaddition in a click ligation reaction [84]. In follow-up studies, hG4 were investigated in vitro and in vivo (in HT-1080 and HeLa cells). The authors used an oligonucleotide model as well as labelling of telomeric RNA sequences to detect the G4s using NMR and circular dichroism spectroscopy, dimethyl sulfate footprinting, and MALDI-TOF mass spectrometry for analysis. These studies support the probability of hG4 formation at telomeres in living cells [4, 83]. It appears that the hG4 structure may have a protective effect, as the replicative capacity of the cells was increased and cellular senescence was reduced [83], indicating that such structures are potentially relevant for cellular physiology.

Although hG4s are a fascinating class of RNA–DNA interactions, more research on their tissue-specific roles, their mechanistic opportunities, and their physiological and pathophysiological importance are needed.

## Conclusion and outlook: the physiological potential of RNA–DNA interactions

**Fig. 5:**
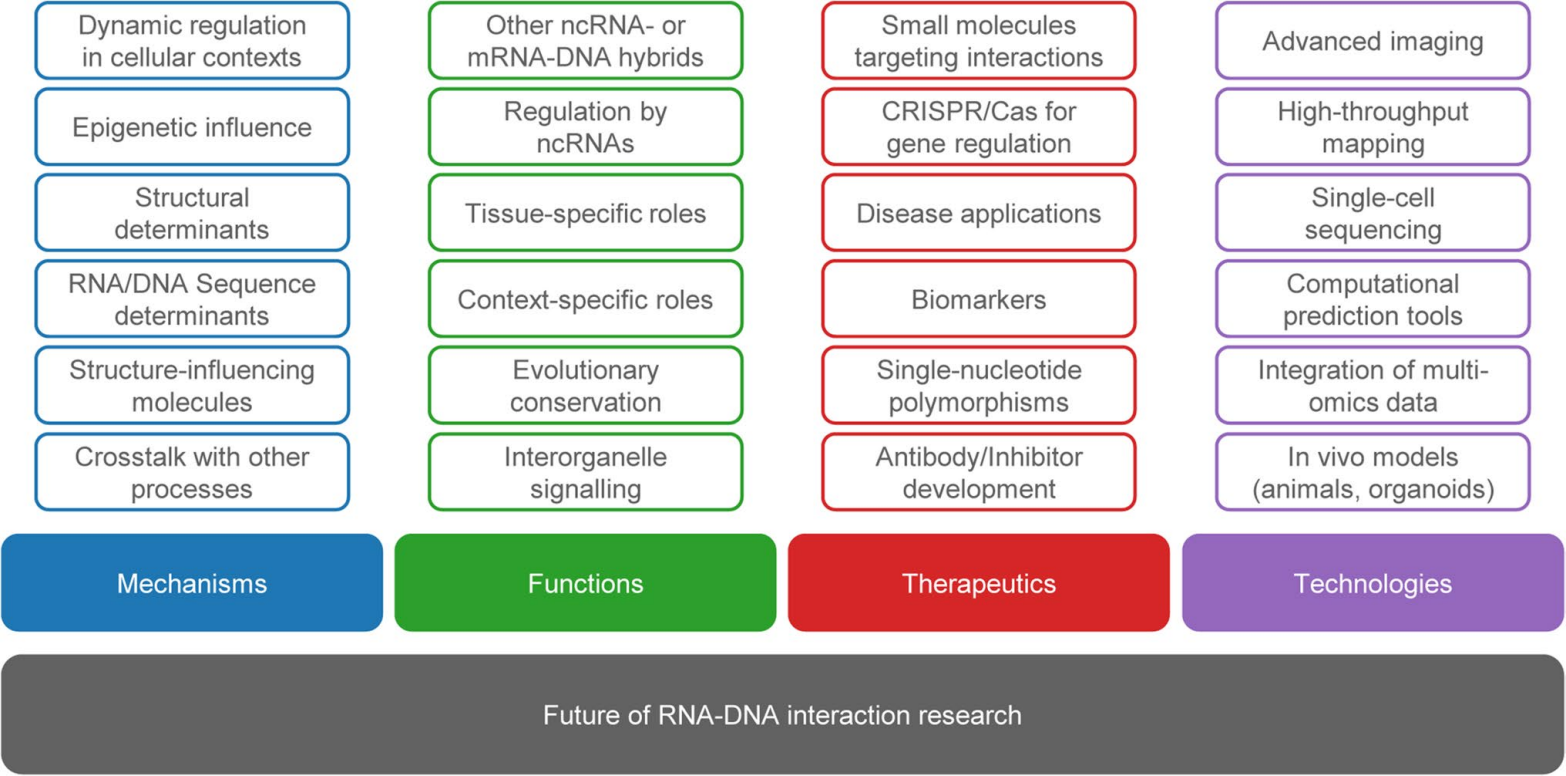
Future directions in RNA–DNA interaction research. Mechanisms to be explored include the dynamic regulation of RNA–DNA interactions in processes such as transcription, replication, and stress responses. Emphasis should be put on the roles of RNA-binding proteins, helicases, and chromatin remodelers, as well as the sequence and structural determinants of hybrid formation and crosstalk with other processes like splicing and DNA repair. Potential functions include the tissue- and context-specific roles of RNA–DNA interactions, while also addressing the emerging roles of non-coding RNAs (e.g., lncRNAs, circRNAs), the evolutionary conservation of these interactions across species and putative implications for interorganelle signaling (e.g., from nucleus to mitochondria, or extracellular communication). Future research on therapeutics will focus on the potential to target RNA–DNA for the treatment of diseases such as cancer, neurodegeneration, and aging. Particular emphasis should be given to the development of small molecules, CRISPR/Cas systems, and biomarkers. Additional unexplored areas are single nucleotide polymorphisms with relevance for RNA-DNA interactions and the development of specific inhibitors and antibodies against these structures. Finally, technologies will be improved in future to study RNA–DNA interactions, including cryo-EM, super-resolution microscopy, genome-wide mapping techniques, computational tools (e.g., prediction via machine learning models, integration of multi-omics), and in vivo models (e.g., animal models, organoids)

RNA–DNA interactions represent a frontier in molecular biology, with their roles in cellular processes such as gene regulation, transcription, chromatin remodeling, and genomic stability becoming increasingly elucidated and thereby their physiological relevance. As research in this field advances, the potential applications of RNA–DNA interactions across therapeutics, biotechnology, and basic research are becoming more apparent (Fig. 5).

One of the most promising applications of RNA–DNA interactions lies in drug discovery. By precisely manipulating these interactions, novel therapeutic strategies can be developed for a variety of diseases, including cancer, neurodegenerative disorders, and viral infections. R-loops, RNA–DNA triplexes, and hG4s provide unique mechanisms for targeting specific gene regions, enabling the development of drugs that can either activate or silence disease-related genes. For instance, compounds designed to stabilize or disrupt R-loops could offer innovative approaches to reactivate tumor suppressor genes or silence oncogenes in cancer. Additionally, synthetic compounds targeting hG4s may serve as alternative telomere stabilizers, though this remains speculative. Furthermore, gene therapy targeting RNA–DNA triplexes could harness these interactions. Interestingly, not only RNA–DNA but also pure DNA triplexes exist [39], offering strategies for applications as DNA oligonucleotides. As such, it may be possible to achieve targeted gene modifications, offering precise ways to correct genetic defects or treat diseases with a genetic basis.

Beyond therapeutics, RNA–DNA interactions hold promise as biomarkers, where the presence of specific RNA–DNA hybrid structures could serve as diagnostic tools for disease detection. Identifying unique RNA–DNA signatures associated with particular diseases would provide a powerful means of early diagnosis, monitoring disease progression, and evaluating treatment efficacy. In biotechnology, RNA–DNA interactions hold vast potential for biosensor development. By utilizing the specificity of RNA–DNA binding, biosensors could be engineered to detect specific DNA sequences or RNA molecules within living cells, providing real-time, in vivo insights into gene expression or cellular activity. Such biosensors could have applications in diagnostics and drug discovery, enabling precise measurement of biomolecular interactions.

RNA–DNA interactions also have the potential to refine and enhance genetic engineering technologies, particularly CRISPR/Cas-based systems. The most famous CRISPR/Cas9 applications, such as the excision or mutation of certain DNA regions or the modulation of gene transcription, relies on RNA–DNA interactions to achieve targeted DNA cleavage or protein effector recruitment. The single-guide RNA (sgRNA), composed of mature crRNA and tracrRNA, binds to complementary DNA sequences, forming an R-loop that facilitates Cas9-mediated double-stranded DNA cleavage [36, 37, 55, 70, 79] (Fig. 2c). By improving the specificity and efficiency of RNA-guided DNA targeting, these interactions could enhance the precision of gene modifications, minimizing off-target effects and increasing the success of therapeutic applications [9]. Furthermore, RNA–DNA interactions could be leveraged in synthetic biology to construct novel genetic circuits and control gene expression in engineered cells or organisms.

From a basic research perspective, understanding RNA–DNA interactions provides key insights into the mechanisms of gene regulation. By decoding how RNA and DNA interact at a molecular level, we can uncover new layers of gene regulatory networks that govern cellular processes such as differentiation, stress response, and aging. Additionally, due to the generally lower sequence conservation of RNAs compared to proteins [65], the conservation of RNA–DNA interactions, particularly of lncRNAs, remains unexplored so far and is inevitable to study disease-relevant animal models. Such studies could also facilitate research into interkingdom RNA–DNA interactions, offering a more flexible framework for understanding cross-species regulatory mechanisms [6].

Recent advances have also highlighted the role of RNA in organizing chromatin and regulating the three-dimensional architecture of the genome [44, 48, 56]. Understanding how RNA–DNA hybrids contribute to the spatial arrangement of chromatin in the nucleus could transform our knowledge of genome function and the regulation of gene expression in different cell types and during various stages of development. Also, improved bioinformatic tools designed to predict RNA–DNA interactions within living cells will be essential for analyzing their impact on gene expression and identifying new regulatory mechanisms [74].

In summary, RNA–DNA interactions represent a rich area of research with immense potential for cellular physiology. Continued interdisciplinary research in this area will not only deepen our biological knowledge but also pave the way for groundbreaking applications that could revolutionize medicine, biotechnology, and basic science.

## Data Availability

No datasets were generated or analysed during the current study.

## Declarations

### Consentfor publication

All authors provide consent for the publication of the manuscript detailed above.
